# Perceptions on use of home telemonitoring in patients with long term conditions – concordance with the Health Information Technology Acceptance Model: a qualitative collective case study

**DOI:** 10.1186/s12911-017-0486-5

**Published:** 2017-06-26

**Authors:** Jo B. Middlemass, Jolien Vos, A. Niroshan Siriwardena

**Affiliations:** 1 Community and Health Research Unit (CaHRU), University of Lincoln, Brayford Campus, Lincoln, LN6 7TS UK; 2 UCL Interaction Centre (UCLIC) University College London, 66-72 Gower Street, London, WC1E 6EA UK

**Keywords:** Heath information technology acceptance model, Long term conditions, Older people

## Abstract

**Background:**

Health information technology (HIT) may be used to improve care for increasing numbers of older people with long term conditions (LTCs) who make high demands on health and social care services. Despite its potential benefits for reducing disease exacerbations and hospitalisations, HIT home monitoring is not always accepted by patients. Using the Health Information Technology Acceptance Model (HITAM) this qualitative study examined the usefulness of the model for understanding acceptance of HIT in older people (≥60 years) participating in a RCT for older people with Chronic Obstructive Pulmonary Disease (COPD) and associated heart diseases (CHROMED).

**Methods:**

An instrumental, collective case study design was used with qualitative interviews of patients in the intervention arm of CHROMED. These were conducted at two time points, one shortly after installation of equipment and again at the end of (or withdrawal from) the study. We used Framework Analysis to examine how well the HITAM accounted for the data.

**Results:**

Participants included 21 patients aged between 60–99 years and their partners or relatives where applicable. Additional concepts for the HITAM for older people included: concerns regarding health professional access and attachment; heightened illness anxiety and desire to avoid continuation of the ‘sick-role’. In the technology zone, HIT self-efficacy was associated with good organisational processes and informal support; while ease of use was connected to equipment design being suitable for older people. HIT perceived usefulness was related to establishing trends in health status, detecting early signs of infection and potential to self-manage. Due to limited feedback to users opportunities to self-manage were reduced.

**Conclusions:**

HITAM helped understand the likelihood that older people with LTCs would use HIT, but did not explain how this might result in improved self-management. In order to increase HIT acceptance among older people, equipment design and organisational factors need to be considered.

**Trial registration:**

ClinicalTrials.gov Identifier: NCT01960907 October 9 2013 (retrospectively registered) Clinical tRials fOr elderly patients with MultiplE Disease (CHROMED). Start date October 2012, end date March 2016. Date of enrolment of the first participant was February 2013.

**Electronic supplementary material:**

The online version of this article (doi:10.1186/s12911-017-0486-5) contains supplementary material, which is available to authorized users.

## Background

Health information technology (HIT) commonly known as telemonitoring, telehealth or telemedicine is a form of non-invasive, remote, home monitoring of patients’ clinical signs and symptoms [1] used to improve the care and management of people with chronic LTCs, many of whom are aged over 60 years. While the evidence for HIT is mixed, it has been associated with reduced mortality in patients with LTCs including diabetes, COPD [2] and Chronic Heart Failure (CHF) [1, 3, 4]. There is also some evidence that HIT home monitoring may be effective in reducing disease exacerbations [5, 6] with a consequential reduction in hospitalisations in patients with LTCs including COPD [1, 5, 6] and CHF [3], and diminished costs mainly due to reduced hospitalisations [1]. Other studies have not shown HIT home monitoring to be a cost effective addition to usual treatment. There is also mixed evidence for improved quality of life with some studies showing a positive benefit [5, 6], but others [7] including the Whole System Demonstrator (WSD) study [8] showing no improvement in quality of life.

It has been argued that the full benefit of HIT depends on the fit between technology design, the patient and their clinical needs [9]. HIT is not always accepted by patients for a variety of reasons including poor device usability; insufficient training on how to use the technology; lack of computer skills and low self-efficacy [10]; worries about using technology; [11, 12] complicated data transfer procedures [13] and false alarms [11]. Others declined or dropped out as they were reluctant to use the equipment every day [12]. Patients’ concerns in previous studies also include a preference for face-to-face health professional contact rather than HIT [11, 14, 15].

**Fig. 1:**
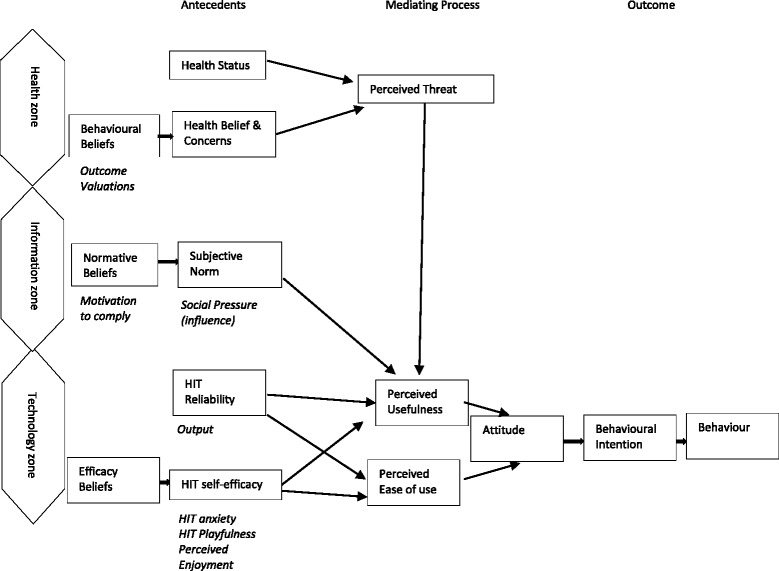
HITAM (after Kim and Park, 2012)

The use of IT in the health care setting is increasing, but adoption is still challenging. In order to understand and introduce HIT, a number of behavioural models and models of innovation acceptance have been studied and applied to the acceptance of technology. One of the most prominent is the Technology Acceptance Model (TAM) [16]. Based on the Theory of Reasoned Action (TRA) [17, 18], the model proposed to address why users accept or reject HIT. The TRA considers behavioural beliefs and attitudes including beliefs of how others would view their behaviour and the motivation of the individual to comply with others’ expectations. These beliefs are thought to lead to behavioural intentions and to behaviour change. The Health Information Technology Acceptance Model (HITAM) is a revised version of the TAM applied to HIT [19]. See Fig. 1. Additional antecedents (precursors) and mediating (facilitating) variables drawn from the Health Belief Model [20] and Theory of Planned Behaviour [21] were added to the hypothetical model. The HITAM was designed to show how users accept and use technology. It Includes 11 determinants influencing attitude towards health technology [19]. There are three zones: health, information and technology zones and three main elements including behavioural beliefs (health beliefs and concerns); normative beliefs (a person’s motivation to comply with using the equipment) and efficacy beliefs (how confident the person is to use the equipment and ease of use; and perceived usefulness of the equipment). The HITAM was chosen as it represents an individual’s behavioural intention to use health technology related to their beliefs and attitudes. Health technology is likely to be of increasing importance in the future, particularly in older people, so it is important to explore how the HITAM applies to this group of vulnerable people.

## Methods

### Aim of this study

By exploring patients’ perceptions and experiences of using telemonitoring equipment in their homes and comparing our findings with the HITAM, we aimed to apply the HITAM to home telemonitoring in older patients with LTCs in order to test the model and to see whether it could be used to help increase the adoption of HIT in this age group. To our knowledge this model has not previously been tested with older people.

### Study design and setting

We used a nested qualitative study as part of a multicentre international clinical trial investigating telemonitoring in patients with long term multi-morbidities including COPD and heart disease. An instrumental, collective case study design [22] was utilised to examine the degree of concordance of the data to the HITAM and identify any possible refinements to the model.

The main study, ‘Clinical trials for elderly patients with multiple disease’ (CHROMED), involved a new home telemonitoring system for patients with COPD and chronic heart failure (CHF) or ischaemic heart disease (IHD) [23] trialled in seven European countries: Italy; Estonia; Spain; Sweden; Norway; Slovenia and two sites in the United Kingdom (Lincoln and Liverpool).

### Characteristics of the participants and study processes

For the study site being described (Lincoln), three patients (over the age of 60 years with severe COPD and an associated heart condition; a current or prior history of smoking and an exacerbation in the past year requiring hospitalisation and/or antibiotics) were recruited, to take part in the pilot study. These patients tested the equipment in their own homes over a period of two months to ensure that any technical issues were identified and resolved. We included those in the pilot phase in the interviews as their contribution to the study was to ensure that the equipment worked smoothly and to iron out any problems, both with the ease of use of the equipment and also the daily transfer of clinical data. We felt that they had a valuable contribution to make in understanding the acceptability of the telehealth equipment.

**Fig. 2:**
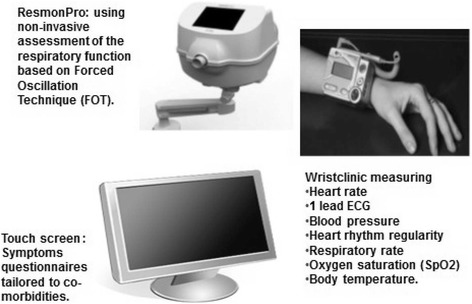
HIT equipment used in the CHROMED Study

Phase B (September 2013 to March 2016) was a full clinical trial with a target of 32 patients at this site who were randomised to either the intervention or control group (i.e. 16 in each group). The intervention group were given the equipment in their own homes with a clinical alert system over a period of nine months and the control group received usual care without HIT. The equipment (See Fig. 2) consisted of a Resmon pro©, designed to measure lung function by calculating airway resistance and reactance through forced oscillation, without the need for patients to forcibly exhale. The Wristclinic was given only to those with confirmed CHF. It measured a wide variety of medical parameters including: heart rate; single lead ECG; blood pressure; heart rhythm regularity; respiratory rate; oxygen saturation (SpO2); and body temperature. Patients also entered daily responses through a computer monitor to a number of symptom questions relating to their illness.

Clinical alerts were created in response to changes in the measures being monitored which could indicate deterioration and predict worsening of the condition, so that remedial action could be taken, either by the patients themselves or by the nurse or other health professional. The hypothesis of the main study was that this would reduce unnecessary hospital admissions and improve quality of life for the patient.

One patient who took part in the pilot study was receiving regular home visits from the heart specialist nurse as she was recovering from acute heart failure. Being on the equipment signalled the withdrawal of the regular nurse visits. None of the remainder had regular (i.e. weekly or monthly) homecare visits from health care professionals.

The selection process for the interviews included those patients who had taken part in the pilot study and all those who were in the Phase B full clinical study who were in the intervention group, including those who subsequently withdrew from the study before four months who were subsequently replaced.

### Data collection

Interviews were conducted two to three days after installation of the telemonitoring equipment and at the end (or withdrawal) from the study to ascertain patients’ perceptions and experiences in order to determine their acceptance of telemonitoring. The interviews were conducted by two experienced qualitative interviewers (JM and JV). The post installation interviews, conducted primarily by telephone, were shorter (approximately 20–30 min). The second interview, at the time the equipment was removed, was conducted at the patient’s home and tended to be longer (approximately 60 min).

### Interview schedules

The interview schedules (see Additional files 1 and 2) contained open-ended questions based on the HITAM elements. The main differences between the two interviews were the post-installation interview included prior use of health and social care services and patients’ familiarity with technology. It further sought information about patients’ understanding, rationale and key influencers for taking part in the study and their perception of the initial assessment and training. The second semi-structured interview focused more on how they experienced the use of the devices in terms of reliability and ease of use; how their relatives responded to their participation in the study and whether using the study equipment had influenced their relationship with their health professionals, and if so how. The second interview was informed by other elements in the study for example number of clinical and technical alerts and hospitalisations.

If the carer lived with the patient using the HIT equipment and was present at the time of the interview then they were invited to take part. It was felt they had a unique insight into the equipment, how it worked for their spouse/partner and in one case father, and the degree to which assistance was required. In addition, for pragmatic reasons, it would have been difficult to exclude them.

We audio-taped the interviews, which were then transcribed verbatim by the research team. To ensure the confidentiality and anonymity of the information, all participants were allocated a pseudonym and a five year age range recorded.

### Data analysis

A Framework approach [24] was chosen as it is systematic, rigorous and a flexible analytical method. Data management and analysis were supported by Nvivo 10 software. The research team (JM, JV and NS) read the transcripts repeatedly to immerse themselves in the data. Two researchers (JM and JV) independently manually coded the interviews line by line, using the HITAM as an initial guide, and using sub-themes and additional open codes to capture all significant and meaningful data fragments. In order to interpret the data, the ongoing coding process started close to the text and moved up to a more abstract level. All identified sub-themes were discussed by the research team until consensus was reached, and relevant quotes tabulated with reference to the full text. The next stage of the analytic process was synthesizing the data by mapping and interpreting. The themes were charted and transferred to a map of the HITAM. Where the theme did not fit the HITAM the research team explored where the additional theme would best fit on an amended model.

Rigour and trustworthiness of the study were ensured through the application of the criteria of credibility, transferability, dependability and confirmability [25]. For example using individual interviews across two time points and across collective case studies, the research team were able to consider similarities and differences across accounts, discuss and double-check the accuracy of data interpretation and provide a detailed description of the research process and context in which this work was undertaken.

## Results

All except one patient allocated to the equipment arm of the study were interviewed. There were 21 patients who participated in total. This was made up of three patients in the pilot phase, and 18 who were in the full clinical trial with the equipment. One patient who was acutely unwell at the time of installation was not fit enough and declined to be interviewed. The total also includes three patients in the equipment group who withdrew early from the full RCT and had to be replaced. All the 21 patients had two interviews, one shortly after the equipment was introduced and the second at the end of the study (or withdrawal). The youngest participant was aged 60 years and the oldest 98 years on entry to the study. Three patients in the intervention arm withdrew from the study after the equipment had been installed. Two did not continue beyond initial training; one was acutely unwell shortly after installation and did not feel able to continue and one said that having the equipment in her home made her feel anxious. The final patient who had been in the study for ten weeks suffered a spinal injury following a fall and her relatives felt she was too unwell to continue in the study after discharge from hospital. Eight carers participated in interviews.

With only one or two exceptions (excluding those who withdrew for health or other reasons) those who took part in the study undertook their daily readings. The main reasons for short periods of non-compliance were hospitalization, or if the equipment was faulty and had to be replaced.

The results are further described in terms of the HITAM elements.

### Health zone

The health zone contains health status and health beliefs and concerns and whether these link to a perceived threat in terms of their perceived severity of ill-health condition. We have defined health status as perceived state of health [26], and health belief in terms of what causes illness; whether it can be treated effectively; who should be involved in the process and whether people believe they are able to take the required remedial action [27].

#### Health status, beliefs and concerns

All patients had severe COPD and at least one other heart related condition which for some included confirmed heart failure. Some patients also had other LTCs such as Crohn’s disease or diabetes that were sometimes of a greater concern to them than the conditions being monitored. In general there was acceptance of the unchanging nature of the chronic medical condition(s) and the inevitability of ageing and (eventual) death.

*“I’m getting older and I’m not going to get any better. I haven’t got young genes to repair everything. So if I can pummel along the way I am, I’ll accept it”.*

Kim and Parks (2012) found that initial awareness and concern about an illness was a facilitator of telehealth acceptance which is in line with the Health Belief Model (incorporated within the HITAM) which was supported by our data.

*P: “You know to me, from the point of view that I’m not allowed oxygen because I haven’t got a blood oxygen level that’s low enough.. But at certain times of the day I believe my blood oxygen level is low enough to warrant it but I’ve never been able to prove it, this equipment might help me either prove or disprove it, I don’t know”.*

In contrast, for other participants’ awareness of severity of their condition increased during telemonitoring.

*I* 1 *:”Looking back over the nine months and when you first came into the study what did you feel your overall health was like? Did you have any concerns?*

*P* 2 *:No I didn’t have any concerns about my health really.*

*I: And how do you feel about your health now?*

*P: It has gone down hasn’t it?*

*PH:* 3 *That thing’s made you more aware hasn’t it?*

*P: It has made me more aware. “*

Another expressed concern was that the introduction of the telemonitoring equipment would lead to the withdrawal of face-to-face input from their health professional.

*P: “But I would hope they would still do their person-to-person contact [and] that they wouldn’t just forget, you’re on a machine that’s it…. It’s alright that they’re looking at machines… but it would be nice, once in a while for them to come and say… you’re doing okay, just the little bit of encouragement.”.*

One patient, who had heightened illness anxiety, found that telemonitoring exacerbated their anxiety to the extent that they withdrew immediately after the equipment was installed.

*P: Yes, I have to say I am one of these people who do worry about things. I do get concerned about myself… and I just thought this is silly. This is reminding me every day, then I should think I wonder what my reading is, how good it is or how bad it is and I thought no, get away from illness you know. Every time as soon I started thinking about it, I started thinking about my illness …*

For another couple, using the telemonitoring equipment only served to remind them of a recent serious life-threatening acute illness. They took part in the feasibility study to test the equipment in gratitude to their Heart Failure specialist nurse, but did not wish to continue with home monitoring. This was because to do so meant to acknowledge to themselves there was a potential high risk of a recurrence.

*PH: “People on that [Chromed devices], they’ve got to have a very high risk factor haven’t they? In other words they are saying that anything could happen to this person’s heart or condition in any period of that nine months, whereas in [wife’s] situation we are hoping that it’s never going to happen again. She’s going to get better, better and better”*

### Information Zone

The information zone contains normative beliefs and subjective norms [18] which would increase a person’s motivation to comply with HIT. In line with this we defined normative belief as an individual’s perception of social pressures or relevant others’ beliefs that they should, or should not, perform HIT, whereas subjective norm is an individual’s own perception about HIT which may be influenced by significant others such as close relatives, friends or health professionals.

We looked at the key influencers to patients taking part in the telemonitoring study and found that both health professionals (HPs) and patients’ significant others were key to participation. One male patient felt that his General Practitioner (GP) had personally recommended him for the study (rather than being identified from a computer search) and he found that very reassuring. If a nurse specialist had been involved with the patient and suggested participation in the study they were much more inclined to do so. Some relatives were reassured that their loved one was being *‘kept a check on’* and this relieved anxiety about their wellbeing, particularly following hospital discharge or frequent illness.

One woman said that when the paperwork arrived she showed it to her close friends (including ex-husband to whom she was still in frequent contact). Her friends said *‘you’ve nothing to lose’* and her ex-husband was very influential saying ‘*you must keep on with it – it’s good’*. She said *‘I think if my very, very close relatives with [ex-husband] and if the GP said it is essential…. I would say I’m definitely going ahead with it.”*

### Technology Zone

This zone contains HIT reliability and HIT self-efficacy leading to perceived usefulness and perceived ease of use. The HITAM defined HIT reliability as ‘output quality and result demonstrability’ [19] and HIT self-efficacy as including ‘HIT anxiety, playfulness, perceived enjoyment and objective usability’ (ibid). We have defined self-efficacy as the perception of ease or difficulty of the particular behaviour incorporating the HITAM elements. The concept of self-efficacy is rooted in Bandura’s social cognitive theory [28]. It refers to the notion that one can successfully execute the behaviour required to produce the outcome. Perceived usefulness in health terms is the capacity to obtain desired outcomes, while perceived ease of use is defined as “the degree to which a person believes that using a particular system would be free from effort” *([29]:320).

#### HIT reliability

We asked patients about their perception of reliability of the equipment. Unreliable technology was linked to poor internet connectivity and data transmission in rural areas and led to the creation of a number of technical alerts. These resulted in the research study nurse telephoning and visiting the patient to ascertain the nature of the problem.

*“PW: … a couple of times it didn’t go through very well, but that was an internet problem..*

*P: Yeah one of them wouldn’t go through at all, the breathing thing wouldn’t go through. The others went through but that didn’t.”*

Towards the end of the first phase of the full RCT, there was the means to send the encrypted data utilising the patient’s own Wi-Fi (where it was available) and this led to a reduced number of technical alerts for non-transmission of data.

Occasionally a clinical alert was triggered because of a  patient’s blood pressure or oxygen saturation levels, which were subsequently checked at the practice and found to be normal and led to the practice questioning the accuracy of the equipment.

The Resmon Pro did malfunction on a few occasions and delays getting replacements or the technician to fit this could affect patients’ perception of reliability.

#### HIT Self-Efficacy

The HITAM suggests that self-efficacy (i.e. self-confidence) in the use of telemonitoring equipment is linked to patients’ familiarity and enjoyment with technology such as mobile phones or computers. We found that acceptance and use of HIT was not particularly linked to prior technological experience. However, initial installation processes were very important in improving self-efficacy. Both the research nurse and the technician undertook the installation visit. Whilst the technician set up the equipment, the nurse went through the simple pictorial instruction manual with the patient who then used the equipment under the direction of the technician and conducted a test run. A number of patients did have a few queries in the early days but, using the instruction manual for guidance where necessary and through repetition, gained experience and confidence. Contact details for the research nurse and technician were highlighted in the manual. For the vast majority, any problems were sorted with prompt follow-up either by telephone or face-to-face.

Those who had early difficulties using the equipment were less likely to continue; for example, one patient who withdrew had a brief spell of feeling dizzy when coming off the equipment for the first time. However, support from partners and relatives was notable in increasing HIT self-efficacy and mitigating initial difficulties. On the first trial run of the breathing equipment (Resmon Pro) one patient did panic and the test was stopped prematurely. However, with the support of her husband, the patient persevered the following day and soon mastered the ability to use the equipment with ease. The support of her husband was crucial and it is unlikely that the patient would have continued without it.

*“The very first time I really got panicked. But then the next day when I did it, it was easier, but I was at the start of a chest infection, which did affect me… It helped my husband stood beside me and was chatting saying yeah you’re doing fine, not long to go, just a little bit of encouragement”*

#### Perceived ease of use (mediating process)

We asked patients how easy it was to use the equipment and found this was linked to individual pieces of equipment and their design features. Different patients found challenges with specific pieces of equipment which affected their HIT self-efficacy i.e. patients could have a high (or low) self-efficacy/ease of use with one piece of equipment but this would not necessarily apply to all.

*P: “…. these two screens are so simple and the writing is so clear and the instructions are so clear …. I think if you came in and you’d never seen a computer before, within half an hour you’d have this mastered.”*

For the CHROMED study the pieces of equipment causing the most challenges were the Resmon Pro breathing machine (a prototype model) and the wrist clinic. Some patients found that the mouthpiece on the Resmon Pro was too hard, large and therefore uncomfortable. A major perceived problem mentioned by most patients was the fact that patients could not see the screen to locate the ‘start the test’ button or to check how many breaths they had taken. Most felt that a second person was required (usually spouse) to help them.

*PH: “The thing we find though, it’s for two people. The screen you actually can’t see when you are breathing, or how many seconds you’ve done. It would have been handy if the screen would have been facing you as you were blowing into it. And you would also know when to start it, which you can’t see.”*

Other issues that affected patients’ ease of use included colour of text and background. The size of some of the equipment was a surprise for some patients whose experience of medical machines led them to believe that they would be much smaller. Conversely, the wristclinic was perceived to be too small for someone with failing eyesight to be able to see with ease.

#### Perceived usefulness (mediating process)

A number of patients felt that perceived usefulness was linked with daily monitoring of the condition in order to establishing trends in their health and detect deterioration or improvements. Patients also felt that HIT had potential to provide early warning of an infection, although one patient stated he felt that he knew his body and that he had developed a chest infection faster than the monitoring indicated.

There was perceived safety in being linked to health professionals and an expectation that the health professional was checking data and ready to act if needed. One participant felt that it was like having a health professional coming in every day.

*“I think it felt like having somebody coming in every day, just checking my sats and everything… I feel more comfortable knowing that somebody’s checking it all the time, you know they’re looking at it every day …I feel as if there’s somebody there, although they’re not here, it just machinery. But I know that the phone can ring if I’m not very well… it’s fantastic.”*

#### Factors affecting perceived usefulness – lack of interactivity

Some participants felt the usefulness of doing their own daily monitoring was to be better informed and to increase their self-understanding of the illness. This was not always fulfilled because of a lack of interactive design features in the HIT, in that patients completed the tests sent electronically but they had no feedback on whether it was normal or otherwise, except if they received a telephone call from their practice in response to a clinical alert. Only patients with had heart failure who had been given the wristclinic could see some results in terms of blood pressure, oxygen saturation levels; temperature etc.

*“..I found it very useful because it helps, you I think, to understand what’s happening. The only thing I do wish they would give you is more of an idea of the results”*

One patient and husband had mixed views on the usefulness of the equipment, particularly the lack of two-way communication exchange with a health professional and missed this. The structured nature of the symptom questions reinforced this feeling of remoteness.

*“You can do it a few days on the run and you’re not getting no [any] response and you think, it feels like a non-entity really…, you think what’s the point?..the questions that are asked are easy and basic, …it’s a straight-forward yes or no answers but you’re not getting no [any] response.”*

Patients wanted some indication of what was happening to them, based on an analysis of the clinical data that were going electronically to the study team. For one male this was important in order promote compliance rather than being left in a void especially in the absence of contact with HPs.

*“I’m in a vacuum.. I’m doing something, I’m sending it off to you, [but] there’s no feedback… You‘d be seeing something for your efforts whereas looking at nothing… I don’t think you should be placed in a vacuum for nine months and say blow into this every day.*”

#### Factors affecting perceived usefulness - appropriateness and handling of clinical alerts

Perceived usefulness of HIT was dependent on clinical alerts being both appropriate (that is relevant and not a false alert) and also leading to a timely response. Each practice handled alerts in a way that best suited the practice’s needs; however some were better than others in terms of adherence to the protocol. In most practices, when responding to a clinical alert, a health professional (GP or nurse) telephoned the patient and dealt with any concerns raised. However, in one practice the receptionist rang the patient and passed any problems onto a health professional, whereas someone else in another branch entered it on the database to close the loop. Because the loop often wasn’t closed in a timely manner there were repeated alerts and repeated phone calls to the patient, which affected their perception of the technology's usefulness. In addition, during the first phase the clinical alert parameters were set to standardised fixed readings leading to a number of false-positive alerts for readings that were ‘normal’ for that patient but outside standard settings, resulting in a number of non-relevant clinical alerts. In the second phase of the RCT there was the ability to set personalised clinical readings for specific patients who had low or high measurements as a norm. This made a difference to the appropriateness of clinical alerts.

There was also some patient confusion when HPs telephoned regarding an alert without stating the reason (in line with the protocol), particularly when the patient had no symptoms. This had the unintended consequence that patients started to record their own readings (where they were able to) to aid communication with the HP.

### Outcomes – Attitude, Behavioural Intention and Behaviour

The final section is about attitude, behavioural intention and actual HIT behaviour. We defined the construct of attitude towards HIT behaviour as an individual's positive or negative evaluation of self-performance using telemonitoring equipment. Attitude is determined by the total set of accessible behavioural beliefs linking the behaviour to various outcomes and is based on the theory of planned behaviour [30]. Behavioural intention is defined as an indication of an individual’s readiness to perform a given behaviour, i.e. intention to use or not use the equipment. It should be noted that behavioural intention does not always lead to actual behaviour.

#### Attitude towards HIT and behavioural intention

It can be seen that patients’ positive attitude toward health telemonitoring is governed by many factors outlined in the HITAM. This was particularly the design including interactive features, ease of use and reliability of the equipment in telemonitoring older people. Also affecting attitude and behavioural intention were the perceived timeliness and usefulness of clinical alerts in terms of treating early signs of infections. However, for those with extreme anxiety about their health HIT may not only result in a negative attitude, but may also have a detrimental effect on their health and wellbeing.

All patients coming into the study had the stated intention of using the equipment for the duration of the study.

#### Actual behaviour change in terms of self-management and changes in health care utilisation

Those who did record their clinical measurements from the wristclinic device did so out of interest (or to respond to any alert phone calls from the HP), but none did anything with them in terms of self-management. As the quote below suggests, patients’ perception of how they felt physically over-rode any perceived changes or abnormalities in the test results.

*P: I kept a note [of clinical results] for pure curiosity… I kept a note of oxygen content and all the rest of that.*

*I: Okay and then what… did you change what you did or how you managed your own condition as a as a result of these?*

*P: No.*

*I: No, so more for you just out of curiosity?*

*P: Curiosity, I mean my condition exists so…if I have a good day I’ll go and cut a tree or do something in the garden …*

Some patients visited the health professional’s office more frequently following a clinical alert because they were notified about a problem they hadn’t been aware of previously. One patient’s wife felt that there was an initial increase in the use of health services to get the condition stabilised.

*PW: “Well his condition has improved because of the equipment because the surgery [health professional’s office] has contacted him and he’s seen the doctor again and again and they’ve come up with something to improve his health.”*

Others felt that as a result of being in the study they had their medical conditions stabilised and as a consequence did not need to visit their GPs so often.

*P: I’ve been less to the surgery… Because I think it’s helped me sort everything out. I’m much better on the medication I’m on now for my blood pressure.”*

A nurse at one surgery told a patient that she would not be reviewing him at the surgery respiratory clinic until the study was over because she felt it was ‘*pointless*’. The patient ascribed the reason for this to be because the clinical alerts were negating the need to be seen pro-actively.

A reduction in home visits was perceived as a possibility, although this was with some reservations in terms of the loss of face-to-face contact this would entail. Although participants were concerned about losing face-to-face contact entirely, some did like the potential of HIT to reduce the number of consultations they would have to attend.

### Summary of the data as applied to HITAM

Table 1 gives the main factors for the acceptance of HIT and also those that would impede either the initial exposure of the ongoing participation in their use of HIT.

**Table 1:** Factors for acceptance and non-acceptance of HIT for older people

Factors for HIT acceptance	Factors for non-acceptance of HIT
*Health zone*	• Health status - acute health issues i.e. feeling very ill.
• Acceptance of chronic nature of illness. • Being chronically ill, but not seriously acutely ill at the time HIT was being introduced.	• Non-acceptance of illness or increased anxiety caused by dwelling on it. • Reinforcement of ‘sick-role’. • Fear of losing health professional input into on-going health care.
*Information zone*	
• Positive affirmation from both health professionals and close relatives.	• Perceived ambivalence particularly by health professionals.
*Technology zone*	
• Use of Wi-fi or good internet SIM card connectivity. • Prompt replacement of faulty equipment. • Installation and follow-up support processes that create patient self-efficacy. • HIT equipment design features suitable for older people which include interactive/feedback features so that patients have the option to use the data to self-manage. • Support (practical/emotional) from patient’s partner/family. • Perception that the data will useful for clinicians and in terms of outcomes (for example picking up on infections early). • Personalised clinical alerts triggers and appropriate handling of clinical alerts.	• Lack of data transfer due to inadequate internet connectivity. • Wide variations between HIT and health professionals’ own clinical devices. • Unreliable equipment and lengthy delays in fixing faults. • Design of HIT equipment not suitable for older people for example font colour and size, equipment and button size. • Perceiving that health professionals were not utilising the data or that it was not useful in early detection of acute illness. • Lack of HIT interactivity/feedback on results limiting ability to increase knowledge of own results and ability to self-manage condition. • Inappropriate handling of clinical alerts in terms of lack of timeliness or relevancy in health terms. • Lack of willingness to undertake daily monitoring.

## Discussion

**Fig. 3:**
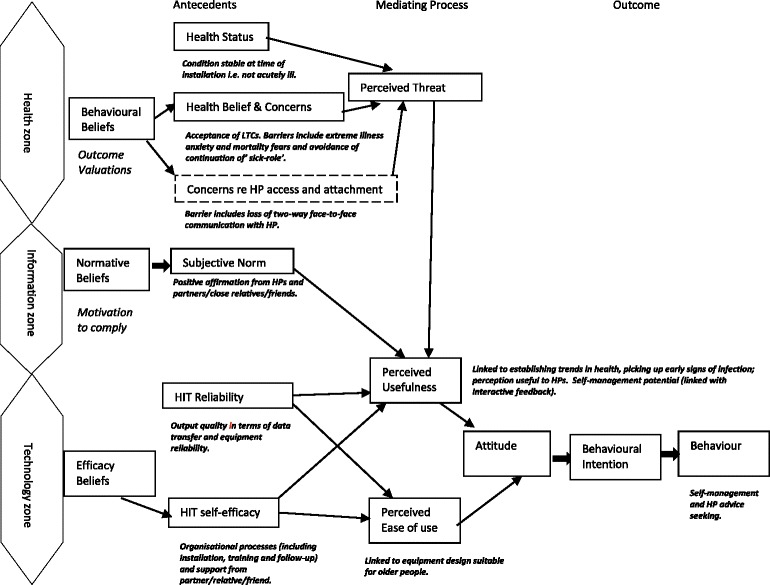
HITAM as applied to older people with LTCs

The HITAM [19] provides a basis for understanding and explaining the acceptance of HIT. We were looking at the model from the perspectives of older people and found that the model did fit to a large extent. However, there were particular factors that needed to be considered when using HIT for older people with LTCs. To our knowledge the HITAM has not yet been used or applied in this way with data from a telemonitoring study. See Fig. 3 for suggested changes to the HITAM for older people with LTCs. Below is a rationale for these changes.

### Health zone

The patient’s immediate health status needs to be considered when planning installation for older people. We found that if the patient was acutely ill at the time of installation (not necessarily related to the nature of the LTC), the chances of non-acceptance of the HIT equipment were more likely.

We found that some patients had stoically accepted the life-restricting (and sometimes life-threatening) limitations of their LTCs and expressed few HIT concerns related to their health, whereas for others, HIT had the potential to positively or negatively impact on both patients and their carers’ anxieties and concerns. Many patients and carers were reassured by the use of HIT, especially following a hospital admission. Kim and Park (2012) also found a significant decrease of family caregiver burden in those families who received HIT intervention in addition to standard care [19]. Conversely we showed that health anxieties if severe, instead became a barrier to use. Many people with LTCs have co-existing clinical anxiety, estimated at approximately 55% of those with COPD [31] and 25–50% among patients with CHF [32, 33]. In our study one patient with extreme illness anxiety became very fearful and withdrew almost immediately after installation. Other RCTs involving telemonitoring have listed anxiety as a reason for withdrawal [34]. This may be reduced by the presence of an informal caregiver in the home [35], a finding which we also identified.

HIT had the potential to reinforce the ‘sick role’ for some patients by continuously reminding them of their diseases. Having increased knowledge through home monitoring makes some patients feel ‘bound to their condition’ preferring instead less information ([36]:8). Others wished to ‘distance themselves from negative connotations of old age, sick and dependence’ [13:6].

How patients perceive HIT will affect their access and attachment to their HP may affect their willingness to participate. One participant felt that it was like having a HP visit every day while another perceived HIT as a potential hindrance to regular face-to-face HP input and communication previously enjoyed. Attachment theory is based on Bowlby theory for children’s attachment with primary caregivers (usually mothers) in order to feel safe and secure [37]. In clinical terms it concerns people’s need to access clinical relationships with health professionals, who are perceived as having expertise to provide security from premature death, especially at times of increased vulnerability, for example during acute serious illness [38] when they are sick or scared [39].

### Information zone

Our study findings confirm the HITAM that normative beliefs and subjective norm, in terms of positive HIT affirmation from HPs and partners/close relatives/friends, were important for older people.

### Technology zone

Whilst our data demonstrated that people who have little or no technological expertise were just as able as those who had to learn how to use the HIT equipment, poor Internet access for those without broadband facilities made using HIT more problematic.

We found initial self-efficacy was related to good organisational processes. We provided Monday to Friday HIT support, but it is noteworthy that two installations on a Friday were subsequently withdrawn. Having no follow-up/support over the weekend may have been a contributory factor as patients struggled to cope on their own for the first few times. Older people often need a ‘positive’ experience with technology to stimulate the uptake of HIT [40]. Czaja (2015) stated that this (positive experience) was more likely to happen if someone showed them how to use technological equipment/devices [41]. Findings from the Whole System Demonstrator study [42] indicated the way technology was first introduced could impact upon perceptions of technical competency. In our study we were fortunate to have expert support when initiating and dealing with electronic monitoring devices and having prompt follow-up support was also vital.

We identified perceived ease of use was connected to having equipment design that was suitable for older people*.* This study supports the notion of designers, patients and researchers working closely together utilising an experience-centred design [43]. Wherton et al. (2015) found participants’ needs were ‘diverse and unique and that technology were rarely fit for purpose’ and suggested co-design workshops with key players including older people to co-produce solutions [44]. Patients and informal carers will be end-users of the HIT, thus incorporating their experiences and opinions in the design process, increases the likelihood that HIT is both effective and meaningful.

We highlighted that perceived usefulness was related to understanding trends in health which patients felt would be useful to their HP in picking up early signs of infection and for self-management potential. One of the main reasons given by patients for participating in the study was to gain a better understanding of their illness. It is interesting that few actually did anything with the results in terms of self-management. However, we do not know whether if the other pieces of equipment (namely the symptom questionnaires or the Resmon Pro breathing device) had been more interactive with some patient feedback, that may have prompted additional self-management or more HP advice seeking. Instead participants preferred to rely on their own perception of daily health in terms of ‘knowing their own body’ and used the results as a communication aid to attempt to second-guess why they were being telephoned in response to a clinical alert. Elwyn et al. (2012) reported that a lack of feedback from telemonitoring systems to patients was a hindrance to effective patient self-management with LTCs [45]. In addition, clarity regarding expectations of HPs’ and patients’ roles and responsibilities would be a helpful first step in encouraging patients to self-manage their condition and increasing awareness of when to contact HPs.

In terms of actual changes in HP involvement our findings showed an early increase in health professional contact to address clinical issues such as raised blood pressure. This was followed by a reduction in reported contacts, with the exception of clinical alerts for potential chest infections. During the second phase of the study the personalisation of clinical data to trigger alerts also led to a reduction in false-positive clinical alerts, further lessening health professional contacts. However, a number of participants were satisfied with their contacts with health professionals, many feeling that they were better ‘served’ during the study than previously when getting an appointment was perceived to be extremely difficult. Previous studies have found variable effects on health service use: Chatwin et al. (2016) found HIT increased usage while others found no reduction [7] or no change [2, 46–50].

### Study limitations

This was a qualitative study of the use of HIT in older people in one rural area. There were limited opportunities for self-management based on the data from the devices. It is not certain whether the inclusion of partners or close relatives of some patients may have affected their responses.

## Conclusion

Our study gives credence to the HITAM as a basis for understanding HIT acceptance in older people and also considers facilitators for HIT users in this age group which should inform HIT designers and implementers.

Our findings support the idea of HIT designers working with older people to ensure that equipment is usable. It is important to identify those for whom HIT would be beneficial, and those for whom it would not be suitable; an assessment of health fears and anxieties and perceptions of whether HIT would reduce or increase these is crucial. Installation processes should be well planned and there needs to be adequate support following installation.

Further research should more fully explore patients’ intentions to self-manage and change behaviour as a result of using HIT. Greater understanding is needed of whether HIT alters patients’ and HPs’ perception of respective roles and responsibilities as well as levels of access and attachment.

HITAM increased understanding  and helped explain whether older people with LTCs would use HIT, but did not help inform whether this would result in improved self-care behaviours.

## Additional files

Additional file 1: CHROMED post installation telephone interview. Interview schedule post-installation (DOCX 17 kb)

Additional file 2: CHROMED end study interview schedule. Interview schedule end of study or withdrawal (DOCX 17 kb)

## Abbreviations

CHF: Chronic Heart Failure
CHROMED: EU funded Clinical trials for elderly patients with multiple disease
COPD: Chronic Obstructive Pulmonary Disease (COPD)
GP: General Practitioner
HIT: Health information technology
HITAM: Health Information Technology Acceptance Model
HP: Health Professional
LTCs: Long term conditions (LTCs)
TAM: Technology Acceptance Model
TRA: Theory of Reasoned Action
WSD: Whole System Demonstrator Study

## Footnotes

1 I = Interviewer.

2 P = Patient

3 PH = Patient’s husband
