# Supplementary material for: Perceptions on use of home telemonitoring in patients with long term conditions – concordance with the Health Information Technology Acceptance Model: a qualitative collective case study
Source: BMC Med Inform Decis Mak. 2017 Jun 26;17:89. doi: 10.1186/s12911-017-0486-5 (PMC5485538; doi:10.1186/s12911-017-0486-5)
Supplement: Supplementary file 1 — CHROMED post installation telephone interview. Interview schedule post-installation (DOCX 17 kb) [file 12911_2017_486_MOESM1_ESM.docx]

*Additional file 1 CHROMED post installation telephone interview*

| **What did you feel your overall health is like?**   - Does it cause you any concerns? - If yes, what are your main concerns? - Do you think the equipment might change this? |
| --- |
| **Can you tell me about what health care and social services you use and how helpful you find these?**   - Do you have a particular GP or nurse that deals with your healthcare? - Have you been into hospital the last year and if so for what reason? |
| **Can you tell me why you agreed to take part in the study?**   - What were your initial expectations? - Did you know what equipment was being delivered? - Was the equipment what you were expecting? If not, how did it differ? - Did you receive sufficient information? If not, what information do you think you should have had? |
| **Before these devices were installed, did you were apprehensive about using technology?** |
| **Do you have a mobile phone, a tablet or do you use a computer?**  How do you get on with these?   - What are your main reasons for their use? - Do you enjoy playing around with these or are they strictly for a restricted purpose? |
| **Have you ever used telehealth equipment before?**   - If so what were your experiences? |
| **How confident were you when the equipment was installed on [date] to perform tasks involved with using the Chromed system?** |
| **How have you got on with the telehealth equipment since [date installation]?**   - What were your initial thoughts when it was installed on [date]? - How do you think about it now you’ve used it over the [weekend]? - How easy/difficult is it to understand the instructions? - If you had any difficulty, what did you find difficult and why? - Has anyone had to help you use the technology the last couple days? If so, who? - Has it entailed doing more or less than you were expecting?   **Do you anticipate any problems with the use of the equipment?**   - If so can you say what these might be? |
| **How useful do you think using the equipment is in helping to keep you well and in picking up on the early signs of a chest infection?**   - What aspects at this early stage do you especially find useful or not useful? - What are your preliminary views on the organisational and technical back-up that the system is supposed to deliver? - What feeling did these past days of use give you regarding how you feel about your condition based on the information from the equipment? |
| **Going forward, do you anticipate any advantages with the use of this equipment?**   - Do you think you will be able to manage your condition better? - How do you feel the telehealth might affect your health care? - Do you think the equipment may alter the healthcare and social care that you currently have? |
| **What do you think your family and friend think of you using the equipment?**  **What do you think health professionals think about you using this equipment?**   - How important is this for you? - Which set of views would be most important in your decision to use the equipment in the end? |
