# Supplementary material for: Perceptions on use of home telemonitoring in patients with long term conditions – concordance with the Health Information Technology Acceptance Model: a qualitative collective case study
Source: BMC Med Inform Decis Mak. 2017 Jun 26;17:89. doi: 10.1186/s12911-017-0486-5 (PMC5485538; doi:10.1186/s12911-017-0486-5)
Supplement: Supplementary file 2 — CHROMED end study interview schedule. Interview schedule end of study or withdrawal (DOCX 17 kb) [file 12911_2017_486_MOESM2_ESM.docx]

*Additional file 2 CHROMED post study interview schedule*

| **What do you feel your overall health is like now and has this changed over the past 9 months?**   - Does it cause you any concerns? - If yes, what are your main concerns? - Did you think the equipment had any impact on this? |
| --- |
| **Can you tell me about what health care and social services you use and whether these have changed over the last 9 months?**   - Do you have a particular GP or nurse that deals with your healthcare? - Have you been into hospital the last nine months and if so for what reason? - Do you think the equipment altered the healthcare and social care that you receive? |
| **Did you have any clinical alerts during the nine months, and if so, how did you feel they were managed?**   - Did you receive a telephone call from the practice? What happened as a result? - How did you feel about the alert process? |
| **How useful do you think using the equipment is in helping to keep you well?**   - If useful, in what way? If not, why not? - What aspects did you especially find useful or not useful? - How you feel about your condition based on the information from the equipment? |
| **Did you make any changes in terms of self-managing your conditions as a result of the information?**   - How does having the equipment over the last 9 months make you feel about managing your condition: more, or less confident, or about the same? - Can you explain why this is this case? |
| **How have you got on with the equipment?**  For each piece of equipment:   - How easy was it to understand the instructions? - If you had any difficulty, what did you find difficult and why? - Has it entailed doing more or less than you were expecting? |
| **Were there any problems with the use of the equipment?**   - How reliable was the equipment to use over the 9 months? - Were there any issues with the internet connection? - If you had any problems how easy was it to get these resolved? |
| **What do you think your family and friends think of you being in the study and using the equipment?**   - What sort of things have they said to you?   **What do you think health professionals think about you using this equipment?**   - How important is this for you? - Which set of views would be most important in your decision to use the equipment in the end? |
